# Comparing Three Methods of Selecting Training Samples in Supervised Classification of Multispectral Remote Sensing Images

**DOI:** 10.3390/s23208530

**Published:** 2023-10-17

**Authors:** Hongying Zhang, Jinxin He, Shengbo Chen, Ye Zhan, Yanyan Bai, Yujia Qin

**Affiliations:** 1 College of Earth Sciences, Jilin University, Changchun 130061, China; hongyingz21@mails.jlu.edu.cn (H.Z.); yanyanbai21@mails.jlu.edu.cn (Y.B.); qinyj2220@mails.jlu.edu.cn (Y.Q.); 2 College of Geoexploration Science and Technology, Jilin University, Changchun 130061, China; chensb@jlu.edu.cn; 3 Aviation Operations Service College, Aviation University Air Force, Changchun 130021, China; xuzj@jlu.edu.cn

**Keywords:** remote sensing classification, sample selection method, classification model, sample size

## Abstract

Selecting training samples is crucial in remote sensing image classification. In this paper, we selected three images—Sentinel-2, GF-1, and Landsat 8—and employed three methods for selecting training samples: grouping selection, entropy-based selection, and direct selection. We then used the selected training samples to train three supervised classification models—random forest (RF), support-vector machine (SVM), and k-nearest neighbor (KNN)—and evaluated the classification results of the three images. According to the experimental results, the three classification models performed similarly. Compared with the entropy-based method, the grouping selection method achieved higher classification accuracy using fewer samples. In addition, the grouping selection method outperformed the direct selection method with the same number of samples. Therefore, the grouping selection method performed the best. When using the grouping selection method, the image classification accuracy increased with the increase in the number of samples within a certain sample size range.

## 1. Introduction

Multispectral remote sensing images contain a large amount of ground object information, which is encoded in various bands of the image. Ground object information can be quickly extracted from multiple bands. Different ground objects have different spectral characteristics, and similar ground objects have the same or similar spectral characteristics under the same conditions. The same ground object exhibits different radiation energy in different bands, resulting in differences between images obtained from different bands. With the rapid development of remote sensing technology, the information that we obtain from images is becoming increasingly rich and, correspondingly, image information extraction technology is constantly improving. In the field of remote sensing, image classification has always been the focus of research for professionals, who are committed to researching advanced classification methods to improve the accuracy of remote sensing image classification. As a fundamental image processing method, remote sensing image classification is the basis for environmental and socioeconomic applications. It has been widely used in various fields, such as environmental protection, land change monitoring, agricultural planning, water resource analysis, natural disaster detection, biodiversity monitoring, etc. [1].

Remote sensing image classification is a complex process, in which every step is crucial, from the selection of data sources and the design of sample selection schemes to the selection of classification methods and the evaluation of classifier performance. The complexity of the landscape in the study area, the scale of the study area, and economic conditions are also important factors that influence the selection of remote sensing data, the design of classification algorithms, and the quality of classification results [2]. However, many previous studies have focused specifically on advanced classification methods and improving the accuracy of remote sensing image classification [3,4,5,6,7] but paid little attention to the research on the selection methods for training samples in classification algorithms.

The structure of this paper is as follows: In this study, the background and motivation of the research are first introduced, outlining the objective of achieving better classification results in remote sensing imagery by selecting appropriate sample selection methods. The paper provides an overview of the current research status in this area. Subsequently, the study elaborates on the three sample selection methods employed. The data and models used in this research, including Sentinel-2, GF-1, and Landsat 8 remote sensing data, as well as SVM [8], RF [9], and KNN [10] classification models, are then introduced. The design and execution processes of the experiments, encompassing sample selection, model training, and the utilization of remote sensing imagery, are described. In the section presenting the experimental results, a comprehensive analysis and comparison of the classification accuracy achieved through different sample selection methods is presented. Additionally, this study explores the influence of varying sample sizes on classification accuracy under the optimal sample selection method. Finally, the Conclusions and Outlook section summarizes the main findings and contributions of this study, offering recommendations for future research directions.

## 2. Related Works

Samples in remote sensing image classification are mainly used as training data for classification models and as test data to evaluate the accuracy of map products. In supervised classification of multispectral remote sensing images, common classification methods include SVM, RF, and KNN. Training samples are required to train the classifier, and the method of selecting the samples determines the quality of the training samples and has a significant impact on remote sensing image classification and accuracy evaluation. With the development of remote sensing technology, the amount of information contained in remote sensing data is increasing. Many researchers have also begun to pay attention to the impact of samples on classification results. Koreen et al. conducted a study analyzing the effects of input data features on the RF classification algorithm. Their results showed that the RF classification algorithm was highly sensitive to the training dataset, and the selection strategy of specific input variables (i.e., image channels) and training data used in classification had a significant impact on the overall accuracy of image classification [11]. Zhen et al. delineated reference polygons to obtain training and validation data and studied the effects of four selection schemes on the classification accuracy and accuracy estimates obtained from validation data in object-based classification research [12]. J. Corcoran et al. selected training samples using three methods—namely, selecting points interpreted from the field and photographs, fixed windows around points, and image objects intersecting with points—and studied the effects of point- and polygon-based training data on the accuracy of wetland RF classification [13]. Shahriar et al. studied the effects of classifier selection, reference sample size, reference class distribution, and scene heterogeneity on the accuracy of pixel classification. They found that SVM and KNN have a significant advantage in accuracy for edge pixels, and that the class distribution in the training dataset has an impact on the accuracy calculation [14]. Ming et al. investigated the impact of training samples and classifiers on Landsat 8 image classification and found that SVM had the highest classification accuracy. The accuracy of the classifier increased with the increase in the training sample size, providing guidance for the selection of training samples and classifiers [15]. Li et al. researched the effect of sample size on remote sensing classification [16,17,18]. Christopher et al. evaluated four sample selection methods (simple random, proportionate stratified random, disproportionate stratified random, and judicious sampling) and three cross-validation adjustment methods (k-fold, leave-one-out, and the Monte Carlo method) for regional-scale machine learning classification. They trained supervised machine learning algorithms using the selected samples to generate land cover maps of large geographic areas [4]. Additionally, Jin et al. studied the effects of four training sample selection schemes on urban and non-urban binary classification in the Denver metropolitan area of Colorado, including two stratification schemes (spatial and class-specific) and two sample allocation options (proportional area and equal allocation) [19]. Lv et al. proposed a grouping-based sample selection method that applies histogram analysis to select more distinctive samples [20].

These studies indicate that sample selection significantly influences the outcomes of remote sensing image classification. Researchers have explored various factors, such as input data features, strategies for selecting training datasets, acquisition of reference polygons for training and validation data, training data based on points and polygons, classifier selection, reference sample size, reference class distribution, scene heterogeneity, sample size, and sampling and cross-validation adjustment strategies. They have found that these factors have a notable impact on the overall accuracy of image classification. Additionally, researchers have proposed specific sample selection methods. In particular, researchers have employed various sample selection methods, including random sampling, stratified random sampling, disproportionate stratified random sampling, and judicious sampling. They have also investigated different cross-validation adjustment methods, such as k-fold, leave-one-out, and the Monte Carlo method. The choice of these methods produces varying effects in different scenarios. For instance, in the context of regional-scale machine learning classification, selecting appropriate sample selection methods and cross-validation adjustment methods can generate land cover maps for large geographic areas. Furthermore, researchers have focused on classifier selection and observed distinct advantages for specific classifiers such as SVM and KNN in certain situations. These studies provide valuable guidance for remote sensing image classification, enabling researchers to more accurately select samples and classifiers, thereby enhancing the accuracy and reliability of remote sensing image classification.

## 3. Materials and Methods

The process of identifying features in remote sensing images essentially involves transforming the identification problem into sample classification. The quality and representativeness of training samples directly impact the classification results of remote sensing image classifiers. Therefore, it is crucial to quickly and effectively select representative training samples. In this paper, we consider each pixel in the remote sensing images as a sample. Each pixel is composed of multiple bands, and these band data contain abundant information. We chose to use the band data of each pixel as sample data. We chose the grouping method and the “entropy”-based selection method for sample selection. Using Sentinel-2, GF-1, and Landsat 8 remote sensing images, as well as SVM, RF, and KNN classification models, we further analyzed the influence of these selection methods on the classification accuracy of remote sensing images.

### 3.1. Image Data

This experiment primarily utilized three remote sensing images: Sentinel-2, Landsat 8, and GF-1. Table 1 provides specific details about these images, all of which underwent preprocessing steps such as radiometric calibration and atmospheric correction.

### 3.2. Classification Models

SVM, RF, and KNN are widely used land cover classification models in the field of remote sensing. These models exhibit excellent performance and versatility when it comes to handling remote sensing data and addressing land cover classification tasks. They are chosen for land cover classification because of their ability to effectively process various types of remote sensing data, including multispectral, hyperspectral, and remote sensing imagery, and to provide accurate classification results across different land cover scenarios. These models play a crucial role in geographic information systems (GISs), environmental monitoring, land-use planning, resource management, disaster monitoring, and more, offering robust support for decision-making and spatial analysis. Therefore, selecting these models for remote sensing land cover classification is a logical choice, given their proven excellence and broad application prospects in this field.

SVM is a supervised learning algorithm used to find the optimal hyperplane for separating the feature space. It uses support vectors to determine the position of the hyperplane, which are the training samples closest to it. SVM transforms the feature space into a higher-dimensional space using kernel functions, enabling linearly inseparable data to become linearly separable in the new space. There are many types of kernels, and in this experiment, the radial basis function (RBF) kernel [21], commonly used in remote sensing, was used as a baseline for evaluating the performance of new SVM kernels.

RF is a machine learning classifier that leverages multiple decision trees for ensemble learning and improves accuracy by aggregating their classification results. Each decision tree acts as a classifier, and the final classification result is determined by the voting of all classifiers.

KNN is a lazy supervised learning classification algorithm that determines the class of a new sample by finding the k most similar samples. The value of k in the KNN algorithm affects the complexity of the decision boundary, with smaller k leading to complex decision boundaries and larger k improving the model’s generalization ability.

### 3.3. Sample Selection Method

We conducted experiments using the group-based sampling method [20]. The group-based sampling method evaluates the feature distribution of each category by analyzing the histogram of the number of samples in each category, and then it selects training samples from different groups in the histogram. This method considers the heterogeneity of categories and is capable of selecting training samples with more prominent features while excluding mislabeled sample points. Additionally, a method based on “entropy” for selecting training samples was proposed. Through experimental analysis, the optimal sample selection method was determined, enabling supervised classification with a small number of representative samples, thus improving the classification efficiency and accuracy. The following is a detailed introduction to the sample selection methods used:

#### 3.3.1. Group-Based Selection Method

Based on the existing method [20], considering that different land features in the image have varying areas and spectral ranges, we developed a specific calculation method to increase the number of groups. The number of groups for each land feature was calculated based on its spectral range and the number of sample labels. In addition, a variable P was introduced to control the number of selected samples. Below are the specific methods for selecting grouped samples.

Firstly, the remote sensing image to be classified needs to be labeled to select the initial samples. As different features have different proportions in the image, a proportional stratified random sampling method is adopted to ensure the representativeness of the training samples. This means that every pixel in the image has the chance to be selected, and the number of samples selected is determined based on the percentage of each feature’s area in the image to ensure that the number of samples for each feature corresponds to its proportion in the image. At the same time, the number of labeled feature blocks should also match the proportion of each feature in the image.

After obtaining the labeled sample data, a feature distribution map for each feature class is generated based on the gray value of each pixel. The gray value of each pixel is calculated as gray = 0.299 × red + 0.587 × green + 0.114 × blue. Red, green, and blue represent the red, green, and blue bands used in the image, respectively, and the calculation of the gray value equalizes the values of the bands and facilitates data processing.

(1) $${Bins}_{k}=\sqrt[{2}]{\left({band}_{max}-{band}_{min}\right)}\;*\frac{m_{k}}{M}$$

where ${Bins}_{k}$ represents the number of groups of the kth land cover type, ${band}_{max}$ represents the maximum band value in the labeled sample of the kth land cover type, ${band}_{min}$ represents the minimum band value in the labeled sample of the kth land cover type, $m_{k}$ represents the number of labeled samples of the kth land cover type, and M represents the total number of labeled samples of all land cover types. Using Formula (1), each land cover type can be divided into different numbers of groups according to its proportion in the image. Different groups represent different spectral ranges, and these spectral ranges correspond to different variation patterns within the same category. By selecting different numbers of samples from each group, it is possible to consider and capture the internal heterogeneity within the category, thus covering the intraclass heterogeneity.

Next, calculate the number of labeled samples within each group and determine the number of samples selected from each group using the following formula:

(2) $$C_{b_{i}}\;=P*N_{b_{i}}^{2}/T_{K}$$

where $C_{b_{i}}$ represents the number of samples obtained for the ith grouping, $N_{b_{i}}$ represents the ratio of the total number of selected samples to the total number of labeled samples, $T_{k}$ represents the total number of pixels in the ith grouping, and $b_{i}$ represents the total number of pixels of the kth land cover type. Samples of pixels are selected for each group in each land cover type in turn. From this formula, it can be seen that the higher the frequency of $b_{i}$, the more labeled samples are selected from this group. When $b_{i}$ approaches 0, no samples are selected from this group. The samples within this range may be mislabeled compared to most of the samples of this land cover type. Therefore, they are not suitable as characteristic training samples.

**Figure 1:**
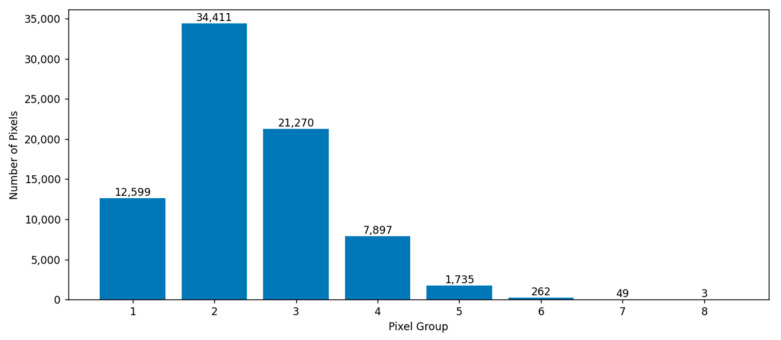
Distribution of woodland features.

Figure 1 shows the characteristic distribution map of the forestland. According to Formula (1), each column represents a group in the histogram. According to Formula (1), the labeled pixels of forestland are divided into eight groups. We need to select a certain number of representative samples from these groups. Formula (2) is used to calculate the number of samples that should be selected for each group. From the histogram, it can be observed that the number of pixels in groups 6–8 is very small, indicating a small N in Formula (2). Therefore, the calculated C approaches 0, which means that no sample points are selected from this interval. This is because pixels within this range may be mislabeled compared to most forestland sample points.

The method of group-based selection takes into account the differences in land features and spectral distributions in remote sensing images. It enables the more accurate selection of representative training samples and ensures that the sample quantity matches the proportion of land categories in the image [20]. This helps to improve the performance and accuracy of classification models.

#### 3.3.2. The Selection Method Based on “Entropy”

In information theory and probability statistics, information entropy is a measure of the uncertainty of a random variable, and it also represents the expected value of information. It is commonly used as a quantifying indicator of information content and serves as a criterion for optimizing system equations or selecting parameters. As the information entropy increases, the number of unstable factors and the level of uncertainty within an object also increase. The magnitude of the information reflects the extent to which uncertainty events are reduced, while the magnitude of information entropy reflects the degree of uncertainty associated with the events.

Drawing upon the definition of information entropy, we propose a sample selection method based on “entropy”. This method employs the “entropy” value to assess whether a sample should be selected. A higher entropy value signifies greater uncertainty and a more significant impact on the overall result, indicating that the sample contains more information. Consequently, we can choose samples with higher entropy values from a multitude of samples as training samples, as they possess more information and are suitable for training purposes. Assuming that the total area of the remote sensing sample to be classified is S and the total number of marked pixels is T, each pixel is calculated according to the following formula:

(3) $$P(s)=-\sum_{k=1}^{n}p(x_{k})\log_{2}p(x_{k})$$

where n represents the number of bands, $x_{k}$ is the value of pixel x in the kth band, and p($x_{k}$) is the probability of x appearing in the kth band. Based on the total number of training samples required, the sample size of each type of land cover is calculated using stratified sampling. The proportion of stratified sampling is the percentage of the area of each type of land cover in the total image area. Samples with higher P(x) values for each type of land cover are selected as training samples, and the higher the P(x) value, the more information it contains.

#### 3.3.3. Direct Sampling Method

In the direct sampling method, we randomly select a portion of samples from the remote sensing image and directly choose a specific number of samples as training samples through the relevant procedure. When compared to the group-based sampling method, we must ensure that the number of samples selected for each category is the same as the number of samples selected in the group-based sampling method.

## 4. Experiments

**Figure 2:**
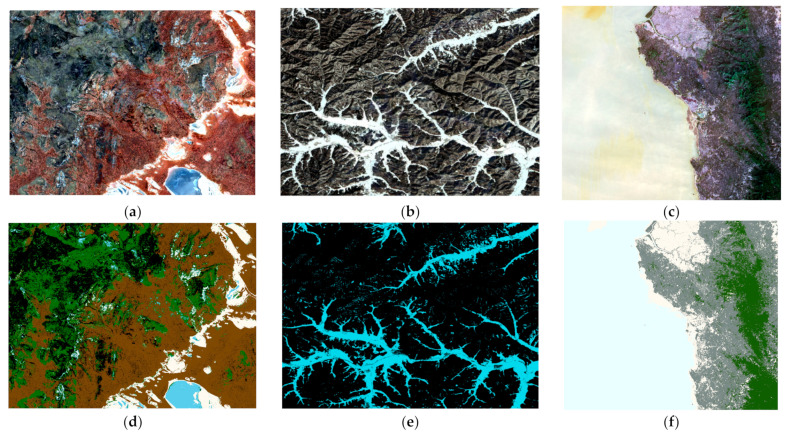
Original images of (**a**) Sentinel-2, (**b**) GF-1, and (**c**) Landsat 8. Classification images for (**d**) Sentinel-2, (**e**) GF-1, and (**f**) Landsat 8.

Three types of remote sensing data—namely, Sentinel-2, GF-1, and Landsat 8—were selected. The remote sensing images were chosen from the red, green, and blue bands. Figure 2 displays both the original images and the classified images of these three datasets. Then, three supervised classification models were chosen, including RF, KNN, and SVM. Sample selection was carried out using the grouping-based sampling method, entropy-based sampling method, and direct sampling method. These three classification models were used to train and classify three images, and the results were compared and analyzed using a test set.

### 4.1. Experimental Steps

#### 4.1.1. Selecting Training Samples

In this experiment, we selected three types of remote sensing data, including Sentinel-2, GF-1, and Landsat 8, as shown in Figure 2. We calculated the land area ratio for each image and collected samples using a stratified sampling method.

Taking Sentinel-2 as an example, the total area of the image was 500,000 square kilometers, of which forests accounted for 50%, grassland accounted for 18%, swamps accounted for 22%, saline–alkali land accounted for 6%, and water bodies accounted for 4%. In Table 2, A, B, C, D, and E represent different land cover categories. We labeled a total of 154,172 sample pixels in the image, including 78,225 forest pixels, 26,844 grassland pixels, 33,643 swamp pixels, 8962 saline–alkali land pixels, and 6498 water body pixels. Table 2 shows the number of pixels of each type of land selected for each image. It can be seen from the table that the number of pixels of each type maintains the area ratio of each land type.

We took the sample sizes presented in Table 1 and divided them into groups for selection using the “entropy” method or directly selecting three methods for screening. When using the grouping method, objects within each region are divided into different groups. The method of selecting samples through grouping helps to identify more representative samples and reduce the overall sample size. The size of the sample can be controlled by adjusting the *p*-value. When the *p*-value was set to 0.5, we selected representative samples from each group and, finally, determined the number of samples for each category, as shown in Table 3. The number of forest samples decreased from 78,225 to 116, grassland from 26,844 to 76, marshland from 33,643 to 150, saline–alkali land from 8962 to 44, and water bodies from 6498 to 23. A total of 409 samples were selected as training samples for model training. It can be seen that the number of samples used was significantly reduced, and that a small number of training samples can reduce the training time of the model. When selecting training samples using the “entropy” method, we calculated the “entropy” value of each pixel and selected a certain number of samples with smaller “entropy” values from each type of object based on the proportion of object area. We trained these training samples using three different models and applied the trained models to the test set to determine their classification accuracy.

#### 4.1.2. Selecting Model Parameters

Many supervised classification models require the selection of appropriate parameters to optimize their performance on specific objectives or datasets. Selecting classifier parameters is a crucial step in the classification process. The optimal parameters cannot be directly determined and require tuning through cross-validation methods. In this experiment, K-fold cross-validation was employed for parameter tuning, with K set to 5. Additionally, the kappa coefficient was used to assess the model parameters. The kappa coefficient is a statistical metric used to assess the performance of classification models, particularly suited for evaluating the accuracy of classification tasks, and is not influenced by class imbalance issues. It considers the consistency between the predicted results of the classification model and the actual observed results by quantifying the model’s performance through comparing correct classifications with random classifications [22]. Table 4 presents the parameter ranges for each classifier, while Table 5 displays the optimal parameters selected through fivefold cross-validation and the model evaluation criterion (kappa coefficient).

We needed to analyze the classification accuracy achievable for each image using the three sample selection methods, evaluate the best sample selection method, and evaluate the influence of sample size under this sample selection method by controlling the number of samples with different labeled sample ratios (i.e., *p*-values) in the total samples.

### 4.2. Analysis of Experimental Results

From Table 6, it can be observed that the different sample selection methods, including direct sampling, entropy-based sampling, and grouping selection, significantly influenced the classification results across different remote sensing images (GF-1, Landsat 8, and Sentinel-2).

Firstly, using the direct sampling method, the classification accuracy for GF-1 ranged from 86% to 89%, while for Landsat 8 it ranged from 31% to 70% and for Sentinel-2 it ranged from 86% to 88%. This method lacks clear selection criteria or strategies. Due to the randomness of the sample selection process and the uncertainty factors, such as different land features and sizes in different images, the classification performance on these three types of images was not satisfactory.

Under the entropy-based sampling method, the classification accuracy for GF-1 ranged from 69% to 88%, while for Landsat 8 it ranged from 79% to 91% and for Sentinel-2 it ranged from 67% to 81%. Compared to direct sampling, the entropy-based method improved the classification accuracy in some cases, but its performance was unstable across different images and models. It did not adapt well to all images and models.

However, the grouping selection method outperformed the others across all remote sensing images and classification models. In GF-1 images, the SVM and KNN classifiers achieved a classification accuracy of 95% using the grouping selection method—significantly higher than the direct sampling and entropy-based methods. In Landsat 8 images, the RF classifier achieved the highest classification accuracy of 94% using the grouping selection method. Similarly, in Sentinel-2 images, the grouping selection method also demonstrated significant improvement, with the SVM classifier’s accuracy reaching over 90%.

The success of the grouping selection method lies in its ability to ensure sample representativeness, thereby enhancing the performance of classification models. It allows the models to adapt better to different images, increasing the accuracy of remote sensing image classification. In contrast, direct sampling and entropy-based methods may lead to non-representative samples, affecting the models’ generalizability and accuracy.

In summary, the grouping selection method exhibits significant advantages in sample selection. It not only enhances the performance of classification models but also increases their generalizability. These models can be applied to different images, achieving high classification accuracy.

Next, let us explore the influence of sample size under the grouping selection method by controlling the number of samples with different labeled sample ratios (*p*-values) and investigate the impact of different sample sizes on the classification accuracy when using the grouping selection method.

For example, Sentinel-2 images were selected to test different *p*-values; nine sets of different numbers of labeled samples were selected, and the samples were used as training samples for three models of KNN, random forest, and SVM classifiers to examine the effects of different sample sizes on the classification accuracy. Table 7 shows the classification accuracies when different *p*-values are taken. From the experimental results in the table, it can be seen that the larger the *p*-value, the higher the classification accuracy. A classification accuracy of 90% can be achieved by all three classification models when the *p*-value is 0.005. The classification accuracy of the RF classifier is improved by 6.1%, that of the KNN classifier is improved by 17.9%, and that of the SVM classifier is improved by 11.8% when the *p*-value is increased from 0.001 to 0.1 and the number of selected labeled samples is increased from 78 to 8311. All of the classification accuracies can reach more than 93%. Therefore, the larger the value of *p* is in the range of 0.001 to 0.1, the larger the number of selected landmark samples and the higher the classification accuracy.

## 5. Conclusions and Outlook

This paper used three methods for selecting training samples: grouping-based sampling, entropy-based sampling, and direct sampling. The KNN, SVM, and RF algorithms were used as classification models. Grouping-based sampling has significant advantages in improving classification accuracy and stability for different image classifications. Additionally, it requires only a small amount of training data, improving the classification efficiency. When using the grouping-based sampling method, the classification accuracy improves as the sample size increases within a certain range. This has practical implications for the classification and identification of land features in remote sensing images. The research findings clearly indicate that incorporating a grouping strategy during the sample selection stage significantly enhances the performance of classification models. This provides effective support for sample selection methods in future remote sensing image processing and analysis.

However, this study also faces certain limitations that need to be considered. Firstly, the experimental results might have been influenced by the choice of datasets and the configuration of the model parameters. In this experiment, we primarily utilized three types of remote sensing images—Sentinel-2, GF-2, and Landsat 8—employing common classification models such as SVM, RF, and KNN. These choices will have had a certain impact on the research outcomes. Secondly, different remote sensing images may exhibit variations in land cover types and differences in land cover distribution and features, potentially affecting the model’s performance. Therefore, it is necessary to further investigate the potential impact of image characteristics on classification results.

In addition, this study only considered three sample selection methods and three classification models. Future research could explore the integration of additional methods and incorporate a broader range of classification models, such as the BP neural network model, to further enhance classification performance. Furthermore, given the crucial role of the spatial distribution characteristics of training samples in determining the final classification accuracy, future studies should comprehensively investigate this aspect to further improve the accuracy of remote sensing image classification.

## Figures and Tables

**Table 1:** Characteristics of the three remote sensing images.

Satellite	Key Features
Sentinel-2	High-resolution multispectral imaging satellite equipped with 13 bands. Primarily used for monitoring terrestrial environments and providing information on vegetation, soil, and coastal conditions.
Landsat 8	Equipped with the Operational Land Imager (OLI) and Thermal Infrared Sensor (TIRS). The OLI has 9 bands with a resolution of 30 m. It is widely used in fields such as global change, agriculture, and water quality.
GF-1	Equipped with high-resolution and multispectral cameras. It can cover large areas with high spatial and temporal resolution.

**Table 2:** Number of total samples for three images.

Image Name	Object A	Object B	Object C	Object D	Object E	Total Sample Size
Sentinel-2	78,225	26,844	33,643	8962	6498	154,172
Landsat 8	118,247	93,162	93,053	24,295	/	328,757
GF-1	249,954	11,202	/	/	/	261,156

**Table 3:** Numbers of training samples for the three images.

Image Name	Object A	Object B	Object C	Object D	Object E	Total Sample Size
Sentinel-2	116	76	150	44	23	409
Landsat 8	364	192	193	89	/	828
GF-1	203	50	/	/	/	253

**Table 4:** The range of model parameter selection.

Classifier	Parameter	Tested Parameter Ranges
SVM(RBF)	C	0.25, 0.50, 1, 2, 4, 8, 16, 32, 64, 128
	gamma	0.001, 0.01, 0.1, 1, 10, 100
RF	num.trees	10, 50, 100, 200
	mtry	1, 3, 5, 7, 9
KNN	K	1, 3, 5, 7, 9

**Table 5:** The optimal parameters of the model under different conditions.

Classification Model		Optimal Parameters Under Different Sample Selection Methods								
	Sample selection methods	Direct sampling method			Group-based selection method			The selection method based on “entropy”		
	Image name	GF-1	Landsat 8	Sentinel-2	GF-1	Landsat 8	Sentinel-2	GF-1	Landsat 8	Sentinel-2
SVM(RBF)	C	8	2	16	8	2	128	0.25	1	0.25
	gamma	0.01	0.001	0.001	0.01	0.001	0.001	0.001	0.001	0.001
	Kappa coefficient	0.7	0.8	0.85	0.76	0.7	0.913	0.6	0.7	0.73
RF	num.trees	200	50	100	100	200	100	10	10	10
	mtry	3	1	3	1	3	1	1	1	1
	Kappa coefficient	0.90	0.83	0.89	0.95	0.94	0.89	0.91	0.86	0.8
KNN	K	3	3	3	5	9	7	1	1	1

**Table 6:** The accuracy of the three classification methods.

Sample Selection Methods	Image Name	Classification Model	Accuracy (%)
Direct sampling method	GF-1	SVM	86
		RF	88
		KNN	89
	Landsat 8	SVM	31
		RF	57
		KNN	70
	Sentinel-2	SVM	87
		RF	86
		KNN	88
Group-based selection method	GF-1	SVM	95
		RF	93
		KNN	95
	Landsat 8	SVM	81
		RF	94
		KNN	93
	Sentinel-2	SVM	90
		RF	93
		KNN	90
The selection method based on “entropy”	GF-1	SVM	69
		RF	88
		KNN	81
	Landsat 8	SVM	79
		RF	91
		KNN	91
	Sentinel-2	SVM	81
		RF	67
		KNN	78

**Table 7:** The classification accuracy at different *p*-values.

The Value of *p*	RF Classification Accuracy (%)	KNN Classification Accuracy (%)	SVM Classification Accuracy (%)
0.001	87.3	76	82
0.003	92.7	89.1	88.4
0.005	93	91.4	90.8
0.007	92.2	90.2	90.6
0.01	92.3	90.4	91.2
0.03	93.2	92.6	92.7
0.05	93.1	93.4	93
0.07	93.3	93.6	93.6
0.1	93.4	93.9	93.8

## Data Availability

Details please see in Supplementary Materials.

## Supplementary Materials

The following supporting information can be downloaded at: https://www.mdpi.com/article/10.3390/s23208530/s1.
